# Activin A Promotes Neuronal Differentiation of Cerebrocortical Neural Progenitor Cells

**DOI:** 10.1371/journal.pone.0043797

**Published:** 2012-08-22

**Authors:** Griselda Rodríguez-Martínez, Anayansi Molina-Hernández, Iván Velasco

**Affiliations:** Instituto de Fisiología Celular - Neurociencias, Universidad Nacional Autónoma de México, México, México; University of Freiburg, Germany

## Abstract

**Background:**

Activin A is a protein that participates principally in reproductive functions. In the adult brain, Activin is neuroprotective, but its role in brain development is still elusive.

**Methodology/Principal Findings:**

We studied if Activin A influences proliferation, differentiation or survival in rat cerebrocortical neural progenitor cells (NPC). After stimulation of NPC with Activin A, phosphorylation and nuclear translocation of Smad 2/3 were induced. In proliferating NPC, Activin produced a significant decrease in cell area and also a discrete increase in the number of neurons in the presence of the mitogen Fibroblast Growth Factor 2. The percentages of cells incorporating BrdU, or positive for the undifferentiated NPC markers Nestin and Sox2, were unchanged after incubation with Activin. In differentiating conditions, continuous treatment with Activin A significantly increased the number of neurons without affecting astroglial differentiation or causing apoptotic death. In cells cultured by extended periods, Activin treatment produced further increases in the proportion of neurons, excluding premature cell cycle exit. In clonal assays, Activin significantly increased neuronal numbers per colony, supporting an instructive role. Activin-induced neurogenesis was dependent on activation of its receptors, since incubation with the type I receptor inhibitor SB431542 or the ligand-trap Follistatin prevented neuronal differentiation. Interestingly, SB431542 or Follistatin by themselves abolished neurogenesis and increased astrogliogenesis, to a similar extent to that induced by Bone Morphogenetic Protein (BMP)4. Co-incubation of these Activin inhibitors with the BMP antagonist Dorsomorphin restored neuronal and astrocytic differentiation to control levels.

**Conclusions:**

Our results show an instructive neuronal effect of Activin A in cortical NPC *in vitro,* pointing out to a relevant role of this cytokine in the specification of NPC towards a neuronal phenotype.

## Introduction

Activins are members of the TGF-β superfamily, that were initially described to participate in stimulating the synthesis of follicle stimulating hormone in pituitary gonadotropes [1]. In addition, Activins are also involved in mesoderm induction in embryonic Xenopus explants [2]. Biologically active Activins consist of homo or heterodimers of two β Activin subunits, giving rise to three proteins: Activin A (βA/βA), Activin B (βB/βB) and Activin AB (βA/βB) [3]. Mature proteins bind to a complex of type I and type II transmembrane receptors with serine/threonine kinase activity. Upon ligand binding, type II receptor phosphorylates type I receptor (also known as Alk4) in its serine/threonine kinase domain, prompting its activation. Type I receptor activation promotes phosphorylation and activation of the proteins Smad 2/3. Once activated, these Smads interact with Smad 4, and together, translocate to the nucleus, where they can directly bind to DNA, or associate with other transcription factors to modulate target gene expression [4].

Activins regulate multiple cellular functions as proliferation, differentiation and cell death in different cell types [5]. In undifferentiated pluripotent P19 embryonal carcinoma cells Activin promotes proliferation [6]. In the case of neurons, it can act as a neurotrophic factor for cultured hippocampal neurons [7], or even as a neuroprotective agent, since it prevents excitotoxic death in mice injected with kainic acid in the hippocampus [8]. Regarding differentiation, Activin A inhibits the retinoic acid-induced neuronal induction of murine P19 cells and IMR 32 neuroblastoma cells, as well as the low-serum-induced neuronal differentiation of GOTO neuroblastoma cells [6]. Treatment of the neuronal-producing subpopulation of the human neuroblastoma cell line SK-N-SH with Activin A causes a dramatic neurite outgrowth, and increases the expression of neuronal markers [9]. Activin A favors the phenotypic markers of cultured hippocampal neurons: it suppressed the emergence of GABAergic interneurons, and increased the number of dentate granule cells, whereas co-treatment with the extracellular Activin antagonist Follistatin, completely abolished these effects [10].

Activins knockout mice have reduced reproductive functions, and although they did not present overt brain alterations [11], [12], craniofacial defects, including cleft palate and loss of whiskers and teeth, were described. In recent years, several reports about Activin signaling components suggest a potential role for Activin A in CNS differentiation and function. During brain development, Activin A mRNA is detected rostrolaterally in the developing cortex, and dorsally in primordial striatum at embryonic day (E)15.5–16. As development progresses, Activin A expression is found enriched at E17 in neurons of the mature deep layers of the cerebral cortex [13]. Similar to Activin A expression, Activin type II receptors (ActRII) are expressed in forebrain regions during E13–E20 [14]. Transgenic mice expressing Follistatin after 2 weeks of age, exhibit enhanced anxiety, as well as a decrease in the survival of newly formed neurons in the adult hippocampus [15].

However, the effects of Activin A in neuronal differentiation during development are still elusive. In order to unravel Activin actions, we analyzed its effects on NPC. These cells are broadly used as a model for studying molecules involved in proliferation and differentiation, because under defined conditions, they undergo controlled cell division and reproducible proportions of terminally differentiated progeny [16]. We measured Smad pathway activation by Activin A binding to its receptors. Different parameters relevant for NPC such as self-renewal, proliferation and expression of molecular markers of neuronal and astrocytic differentiation were also monitored. The results obtained in the present work show that continuous treatment with Activin A increases neuronal number, without altering glial population. These effects were not related to an early cell cycle exit, nor apoptotic cell death. The increases in neuronal differentiation induced by Activin A were present in clonal conditions, and were dependent on activation of Activin-responsive receptors. These findings demonstrate that Activin A positively influences neuronal differentiation of cortical NPC.

## Results

### Effects of Activin A on Proliferating Cerebrocortical Neural Progenitor Cells

Neural stem cells self-renew for extended periods of time and are multipotent, generating neurons, astrocytes and oligodendrocytes after differentiation. Cells from the developing rat cerebral cortex were dissociated and kept in culture with FGF2 for 2 passages. During proliferation, these cells express Nestin and do not show markers for astroglial or vascular cells, ruling out an initial contamination with differentiated cells (Figure S1, upper panel). Cells cultured without FGF2 differentiate producing neurons (β-III Tubulin-positive), oligodendrocytes (O4-positive) and astrocytes positive for Glial Fibrillary Acidic Protein (GFAP; Figure S1, lower panel). These cortical cultures contain a mixture of neural stem cells with progenitors, so we use the term NPC for this undifferentiated neural population that can generate neurons and glial cells.

**Figure 1:**
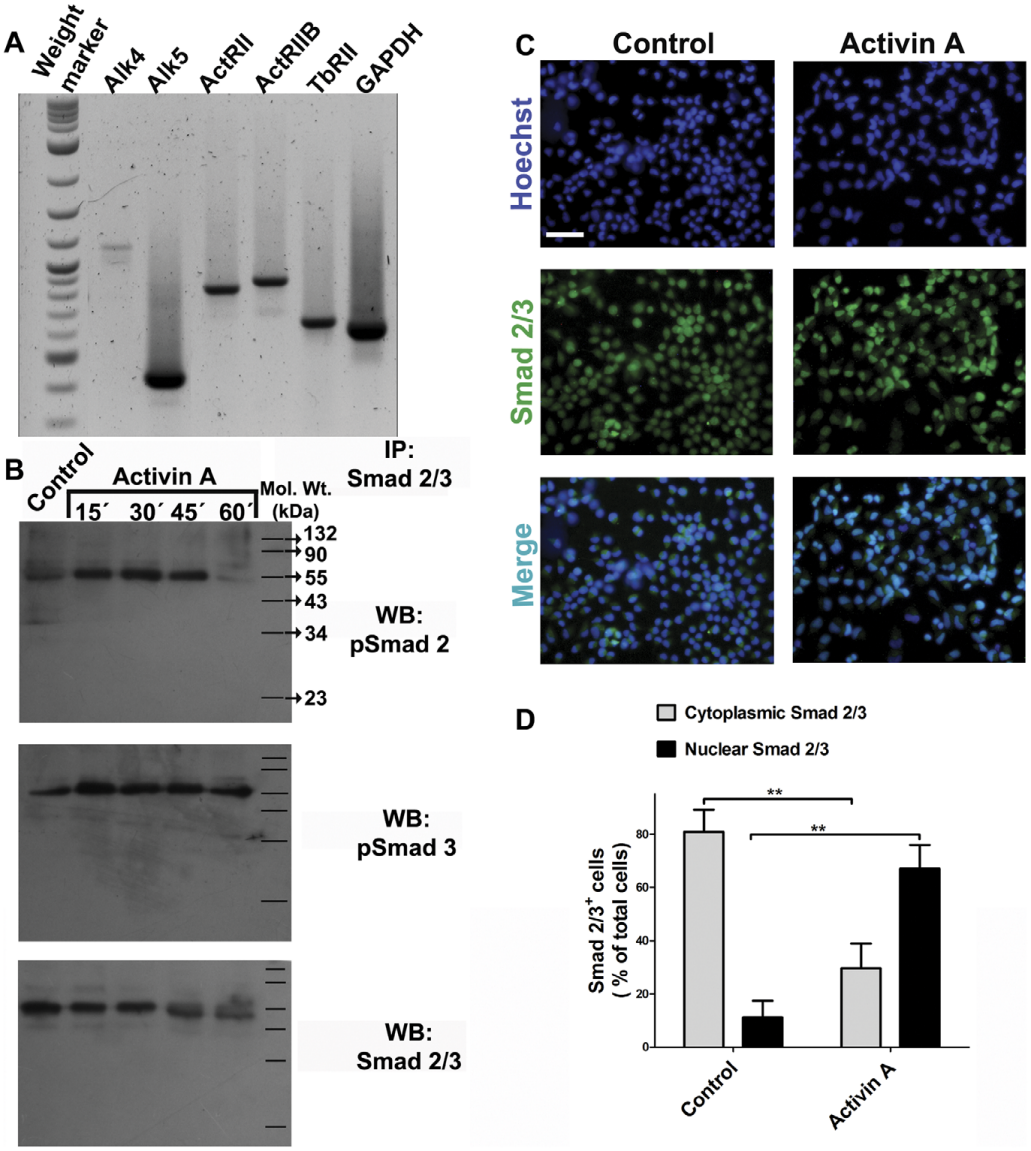
Cerebrocortical neural progenitor cells are responsive to addition of Activin A. Passage 2 neural progenitor cells (NPC) were obtained from E14 rat embryos and kept in proliferation with FGF2. **A:** RNA was isolated from these cultures to perform RT-PCR to detect Activin and TGF-β type I and type II receptors. **B:** Cells were stimulated with 3 ng/ml Activin A at the indicated times. Protein from each sample was obtained and immunoprecipitated with anti-Smad 2/3 antibodies, followed by immunoblot detection of phosphorylated (p)Smad 2, pSmad 3 and total Smad 2/3. The molecular weight (Mol. Wt.) markers are indicated on the right side. **C:** Immunocytochemistry for Smad 2/3 in cells either untreated or treated during 30 minutes with Activin A. **D:** Quantification of nuclear translocation of Smad 2/3 from 10 random fields. The values are represented as mean ±S.D. expressed as the percent of total cells (detected by Hoechst staining in blue) with predominant nuclear staining for Smad 2/3 (green label). **p<0.01 versus untreated condition. Scale bar = 50 µm.

Although only results with 3 ng/ml Activin A are reported, we also performed stimulation of NPC with 10 or 30 ng/ml of Activin A, and found essentially the same results in all tested parameters (data not shown). Previous reports have shown that Activin A type I and type II receptors are expressed in E14 cortex, as well as in NPC cultures derived from the cortex of E14 rat embryos [17]. First, we wanted to determine if NPC growing in the presence of FGF2 express type I and type II Activin receptors. Figure 1A shows that these cells express transcripts of Alk4, as well as those for ActRII and ActRIIB, indicating that cortical NPC might be responsive to Activin A. In addition, NPC also express type I and type II receptors for TGF-β (Alk5 and TbRII, respectively, Figure 1A) and BMP signaling ( [17] and data not shown).

Once Activin A binds to its receptors, type I receptor phosphorylates Smad 2/3 proteins. Then, phosphorylated Smad 2/3 binds to Smad 4 and forms a heterocomplex that translocates into the nucleus, where it regulates gene transcription [18]. In order to assess whether Activin A activates Smad signaling in NPC, we stimulated cells with Activin A and analyzed the phosphorylation status of Smad 2/3 after its immunoprecipitation (IP) with specific antibodies; we also monitored the nuclear accumulation of Smad 2/3 proteins by immunocytochemistry after addition of Activin A. Smad 2 reached their maximum phosphorylation levels 30 minutes after Activin stimulation (Figure 1B). Similarly, Smad 3 phosphorylation increased after 15 minutes and remained elevated for 1 h (Figure 1B). Immunocytochemistry with antibodies that recognized Smad 2/3 in NPC demonstrated that addition of Activin A promotes a significant nuclear accumulation of Smad 2/3 proteins (Figure 1C and 1D), indicating that, in addition to induce Smad 2/3 phosphorylation, Activin A is also able to prompt Smad 2/3 translocation to the nucleus. These results show that cortical NPC are responsive to Activin stimulation.

**Figure 2:**
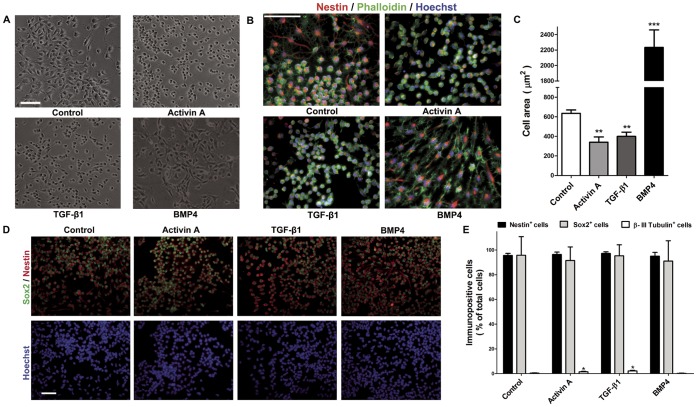
Activin A affects NPC morphology and increases the number of neurons during proliferation. Cells were kept in N2 medium with 10 ng/ml FGF2 during 4 days and treated with the indicated concentration of each cytokine every other day. **A:** Representative microphotographs of cells treated with Activin A, TGF-β1 and BMP4. **B:** Representative images of NPC stained with Phalloidin (green), Nestin (red), and nuclear detection by Hoechst (blue), showing that treatment with TGF-β1 and Activin A caused a decrease in the number of cell extension, as well as reduction in cell body area, relative to control conditions. In contrast, BMP4 produced cell flattening and expansion in cell area. **C:** Cell area quantification after Phalloidin staining of control and cytokine-treated cells, made in duplicate from ten fields taken in three independent experiments. Treatment with either Activin A or TGF-β1 caused a 50% reduction in cell area, relative to control cells. BMP4 produced a three-fold increase in cell area. **D:** Representative images of NPC immunostained with Sox2 (green), Nestin (red) and nuclear detection by Hoechst (blue). Treatment with the cytokines did not change the proportion of cells immuno-posititve for such markers (ANOVA followed by Student-Newman-Keuls test). **E:** Quantification of the percentage of immuno-positive cells for the indicated markers, relative to total cell number, after the indicated treatment. Activin A or TGF-β1 treatment caused discrete but significant increases in neuronal percentages, when compared with control cultures (see Figure S2); however, these raises did not affect overall the percentage of cells expressing the NPC markers Nestin and Sox2. Results are mean ±S.D expressed as the percent of total cells stained with Hoechst. *p<0.05, **p<0.01 and ***p<0.001 versus control condition. Scale bar = 50 µm.

NPC grow with a defined shape and size. In order to investigate if Activin A modifies such parameters, NPC growing with 10 ng/ml FGF2 were treated with 3 ng/ml Activin A. We also stimulated proliferating NPC with 0.5 ng/ml TGF-β1 or with 5 ng/ml BMP4, because their effects are well-described [19], [20], [21], [22], [23], [24], [25]. Control cells in the presence of FGF2 typically show 2 or 3 processes and a round soma under phase contrast microscopy (Figure 2A). Activin A and TGF-β1 had very similar effects, causing a decrease in cell soma size, as well as a decrease in the number of processes. BMP4, on the other hand, caused cell widening/flattening, and considerably augmented the size of cells (Figure 2A). In order to quantify such differences, we performed polymerized Actin staining with Phalloidin coupled to Alexa 488 and subsequent immunostaining with Nestin antibodies (Figure 2B). Quantification of cell area was made by marking the cell borders in digital images obtained for control and experimental conditions after Phalloidin staining. Activin A and TGF-β1 decreased the number of cell extensions, and significantly reduced cell area (Figure 2C) to about a half of that in control cells. In contrast, treatment with BMP4 produced numerous processes and causes a 3.5-fold increase in cell body area (Figure 2C).

To establish whether these morphological changes were due to an early differentiation of NPC in the presence of FGF2, we studied their phenotype by immunocytochemically detecting the expression of Nestin (component of intermediate filaments characteristic of NPC [26], [27]), Sox2 (transcriptional factor restricted to neural stem cells with multipotent and self-renewal properties [28]), β-III Tubulin (early neuronal marker, identified by TUJ1 antibody) and GFAP (astrocyte marker). The proportion of Nestin^+^ cells was above 95 % in control cultures, and did not change after treatment with Activin A, TGF-β1 or BMP4 (Figure 2D and 2E). We did not observe GFAP immuno-positive cells in all studied conditions (Figure S1 and data not shown); however, there were small, yet significant increases in the number of positive cells for β-III Tubulin after treatment with either 3 ng/ml Activin A or 0.5 ng/ml TGF-β1 (Figure 2E and Figure S2). BMP4, on the other hand, did not change the proportions of neurons positive for β-III Tubulin. The proportion of Sox2^+^ cells was unchanged after addition of Activin A, TGF-β1 or BMP4, consistent with the high proportion of Nestin^+^ cells (Figure 2D and 2E).

**Figure 3:**
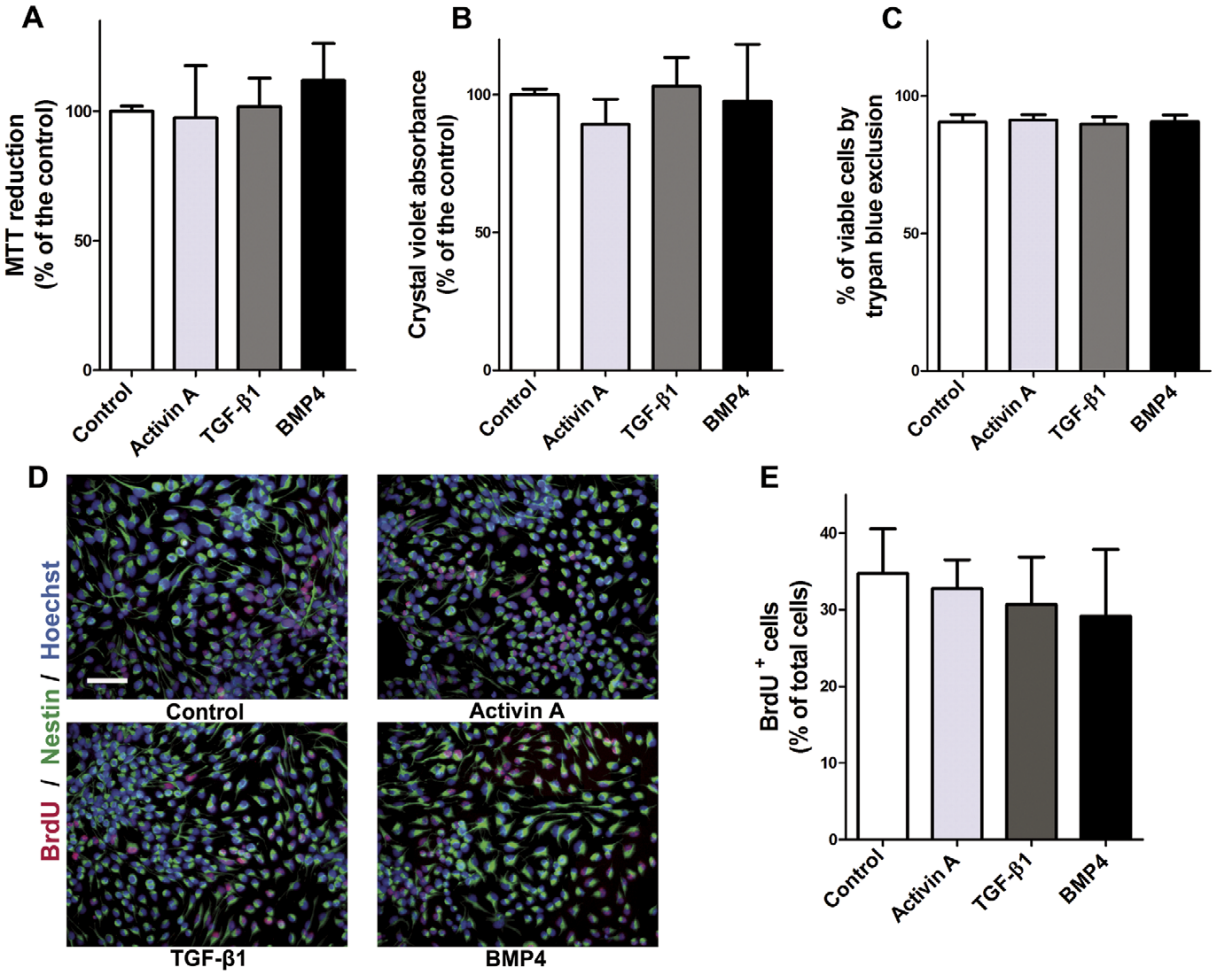
Activin A neither modifies cell number nor BrdU incorporation in proliferating conditions. Cells were kept in N2 medium with 10 ng/ml FGF2 and treated with Activin A, TGF-β1 or BMP4 during 4 days. In the MTT (**A**) or crystal violet (**B**) assays the resulting absorbances in experimental conditions are expressed as percentage of the control condition. **C:** Quantification of viable cells by trypan blue exclusion. Results are expressed as percent of cells that exclude the dye in relation to total cells. **D:** Representative images of the immunocytochemistry for Nestin (green) BrdU (red) and nuclei stained with Hoechst (blue), showing no changes in cell proliferation due to the treatment with the cytokines. **E:** Quantification of BrdU^+^ nuclei. The results are expressed as mean ±S.D. of the percent of total cells stained with Hoechst. Statistical comparisons were made by ANOVA followed by Student-Newman-Keuls test.

Because some TGF-β family members have anti-proliferative cell functions [20], [24], [29], [30], we hypothesized that Activin A could have a potential effect on proliferation of cerebrocortical cultures in the presence of FGF2. To test this idea, we performed MTT, crystal violet and trypan blue exclusion assays to assess the total number of live cells after treatment with Activin A, TGF-β1 or BMP4. The MTT assay showed that treatment of NPC with these growth factors did not modify control values (Figure 3A). Similar results were obtained with the crystal violet assay (Figure 3B). In addition, the proportion of viable cells counted in the trypan blue exclusion assay was unchanged (Figure 3C), implying that proliferation was unaffected by Activin A, TGF-β1 or BMP4. To directly assess changes in proliferation of NPC, we decided to incubate the cells for 3 h with 10 µM BrdU (a thymidine analogue that is incorporated into genomic DNA by cells at S-phase), in order to quantify the percentage of cells that undergoes proliferation. As shown in Figures 3D and 3E, Activin A, TGF-β1 or BMP4 did not induce a decrease in cell proliferation in this assay and NPC continue to divide to levels similar to controls.

### Activin A Increases Neuronal Differentiation of Cerebrocortical Neural Progenitor Cells

**Figure 4:**
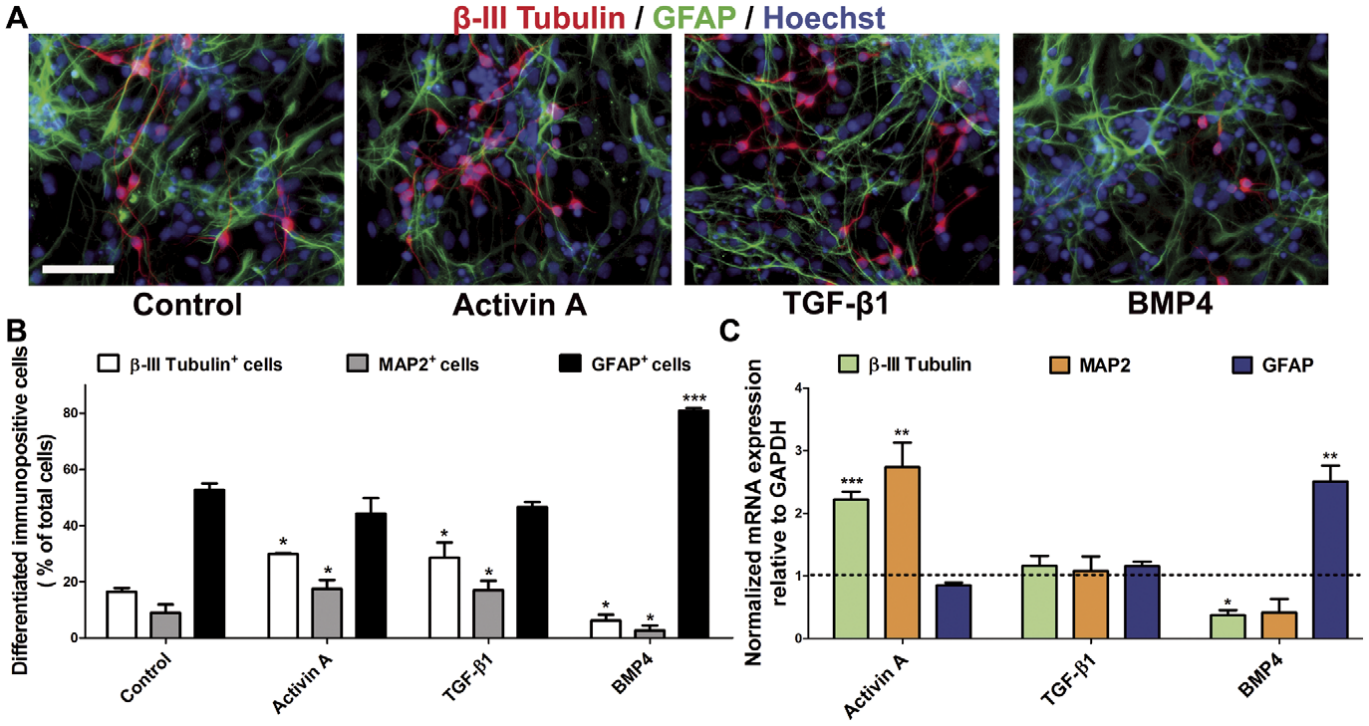
Continuous treatment with Activin increased neuronal differentiation in cultures analyzed 6 days after FGF2 removal. **A:** Representative micrographs of the labeling for β-III Tubulin (red), Glial Fibrillary Acidic Protein (GFAP, green) and nuclear detection by Hoechst (blue), after the indicated treatments, to evaluate the percentage of neuronal or astrocytic cells. **B:** Quantification of the percentage of neuronal (β-III Tubulin- or MAP2-positive) or astrocytic (GFAP-positive) cells relative to total cell number after Activin A, TGF-β1 or BMP4 treatment. Experiments were performed in duplicate, and pictures taken from ten fields from three independent experiments were considered. No cells were found to co-express β-III Tubulin and GFAP. (C) Quantitative RT-PCR from cells continuously treated with Activin A. The values were normalized by the expression level of the housekeeping gene GAPDH and a value of 1 was used for the control condition. Activin A treatment increased twice the level of expression of both neuronal markers tested: β-III Tubulin and MAP2. On the other hand, treatment with BMP4 produced a significant two-fold increase in the expression level for GFAP with a concomitant reduction in β-III Tubulin levels. Results are mean ±S.D. *p<0.05, **p<0.01 and ***p<0.001 versus control condition. Scale bar = 50 µm.

Upon FGF2 removal, NPC undergo differentiation into neurons, astroglia and oligodendroglia (Figure S1, lower panel). Since our results demonstrate that Activin A slightly increases neuronal differentiation of NPC even in the presence of FGF2 (proliferative conditions), we wanted to further explore whether Activin A modified the proportion of differentiated neuronal cells. To test this, we performed immunocytochemistry of cultures maintained for four days in the presence of FGF2 and Activin A, followed by six days of differentiation. When Activin A was omitted from either the proliferation or differentiation medium, no significant changes (ANOVA followed by Student-Newman-Keuls test) in the proportion of neurons (assessed by β-III Tubulin and MAP2 expression) or astrocytes (GFAP^+^ cells) were found (Figure S3). To determine if the presence of Activin A was necessary during the differentiation phase to increase neuronal differentiation, we analyzed NPC that were treated with Activin A in both proliferative and differentiating phases. We found significant increases in the proportion of β-III Tubulin- and MAP2-positive cells after Activin A treatment without modification of astrocyte differentiation (Figure 4A and 4B). As expected, TGF-β1 also augmented β-III Tubulin and MAP2 neurons. BMP4, on the other hand, significantly reduced neuronal differentiation and increased glial cells (Figure 4A and 4B). NPC treated with 5 ng/ml BMP4, in addition to differentiate readily to astrocytes, also significantly induced Smooth Muscle Actin (SMA) expression. In fact, higher concentrations of BMP4 caused progressive increases of SMA-positive cells at the expense of GFAP-positive astrocytes (Figure S4). This SMA-inducing effect of BMP4 has been previously described [17], [22].

Moreover, quantitative RT-PCR from cultures continuously treated with Activin A showed significant inductions on mRNA expression of MAP2 and β-III Tubulin (Figure 4C). Activin A caused a two-fold increase in β-III Tubulin transcripts and 2.5 times more mRNA for MAP2; levels of GFAP expression were unchanged by Activin A (Figure 4C). TGF-β1 did not modify transcriptional levels for these 3 genes relative to control values. BMP4 treatment increased more than 2 times the GFAP mRNA levels, while the levels of the neuronal marker β-III Tubulin was significantly decreased by 50% (Figure 4C).

### Neither Cell Death Nor Cell Cycle Exit Accounts for Activin A Neurogenic Effect

**Figure 5:**
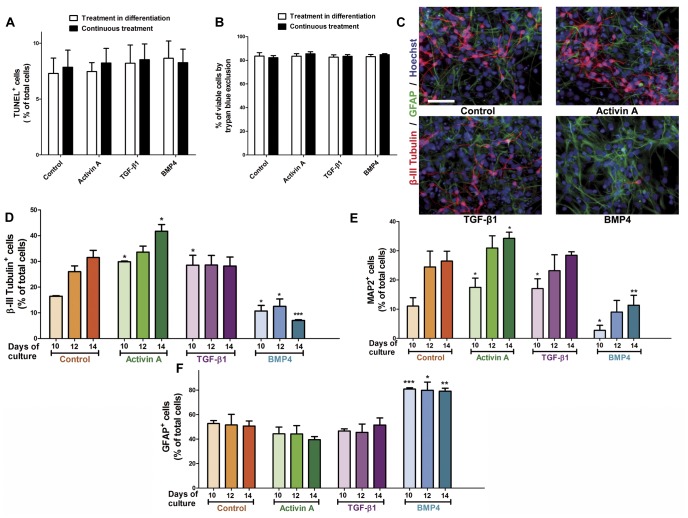
Activin A does not modify cell death nor promotes cell cycle exit. **A:** Cells kept in proliferation for 4 days and 6 days without FGF2 were exposed to Activin A only during differentiation or during both proliferation and differentiation (continuous treatment) did not show significant changes in TUNEL-positive cells. **B:** Quantification of the number of viable cells by trypan blue exclusion assay under the same conditions. Similar to TUNEL assay, no differences were obtained in the number of viable cells treated with the cytokines. Cells were continuously treated with Activin A, TGF-β1 or BMP4 and let to differentiate for 6, 8 or 10 days (10, 12 and 14 days in culture, respectively) to quantify differentiated neurons and astrocytes. **C:** Representative micrographs showing the effects of Activin A, TGF-β1 or BMP4 exposure for 14 days in culture on neuronal (β–III Tubulin^+^) and astrocytic (GFAP^+^) differentiation. Note that the positive neuronal differentiation effect of Activin A prevails after 14 days in culture, whereas the neuronal proportion elicited by TGF-β1 did not differ from control conditions. BMP4 reduced neuronal and augmented glial differentiation. **D–F:** Quantification of the number of β-III Tubulin-, MAP2- or GFAP-positive cells at the indicated days. Neurons (**D** & **E**) increased in control conditions during days 10–14, but the proportion of astrocytes remained stable (**F**). Activin A significantly increased the proportion of neurons at 14 days relative to controls meanwhile TGF-β1 did not augment the proportion of neurons. Astrocytic differentiation was unaffected by Activin A or TGF-β1 treatment. BMP4 caused significant reductions in the neuronal population and enhanced astrocytogenesis at all tested times (**F**). Quantifications were made in duplicate from ten fields in at least 3 independent experiments. Results are shown as mean ±S.D. Student-Newman-Keuls was used as a post-hoc test after one-way ANOVA. *p<0.05, **p<0.01 and ***p<0.001 versus control condition. Scale bar = 50 µm.

The increased neuronal population found after Activin A treatment can be produced by several mechanisms, namely: 1) increased survival of differentiated neurons; 2) promotion in cell cycle exit of neuroblasts; 3) increased neuronal commitment. We therefore analyzed apoptotic cell death in NPC cultures differentiated for 6 days (10 days of continuous treatment). We did not find differences on TUNEL-labeled cells after Activin A, TGF-β1 or BMP4 treatment relative to control conditions (Figure 5A). In addition, neither the proportion of viable cells assessed by trypan blue exclusion assay (Figure 5B) nor the number of total nuclei was changed (Table S1). Having shown that Activin A does not have a neuroprotective role, we went on to test a potential role for Activin A in promoting an early differentiation in NPC. We treated NPC during 4 days of proliferation and either 6, 8 or 10 days of differentiation (10, 12 and 14 days, respectively) with Activin A and analyzed expression of β-III Tubulin, MAP2 and GFAP by immunocytochemistry. Cells treated with Activin A for 10–14 days showed increases in the number of neurons that received continuous treatment with the cytokine (Figures 5C–5F), being consistently significant with treatment during 10 days. Control cells also showed increases in neuronal proportions with increasing times of culture. Neither immuno-positive GFAP^+^ cells (Figure 5F) nor total number of nuclei (Figure S5) were affected by the treatment with Activin A in all days analyzed. TGF-β1 has been reported to have anti-proliferative functions in cortical cells and promote the cell cycle exit [20], [24], [29]. Consistent with these findings, cells that received continuous treatment with 0.5 ng/ml of TGF-β1 showed an increase in the number of β-III Tubulin and MAP2 immuno-positive cells at 10 days of continuous treatment. Interestingly, immature neurons labeled with β-III Tubulin antibodies did not augment after 10 days, whereas MAP2^+^ mature neurons increased its numbers, but without significant differences (ANOVA followed by Student-Newman-Keuls test) with the control at days 12–14 (Figure 5C–5E). BMP4 caused significant diminutions on β-III Tubulin^+^ and MAP2^+^ neurons at all tested times and also caused significant increases on astrocytic differentiation after 10–14 days (Figure 5C–5F).

### Activin A Increases Neurogenesis in Clonal Conditions

**Figure 6:**
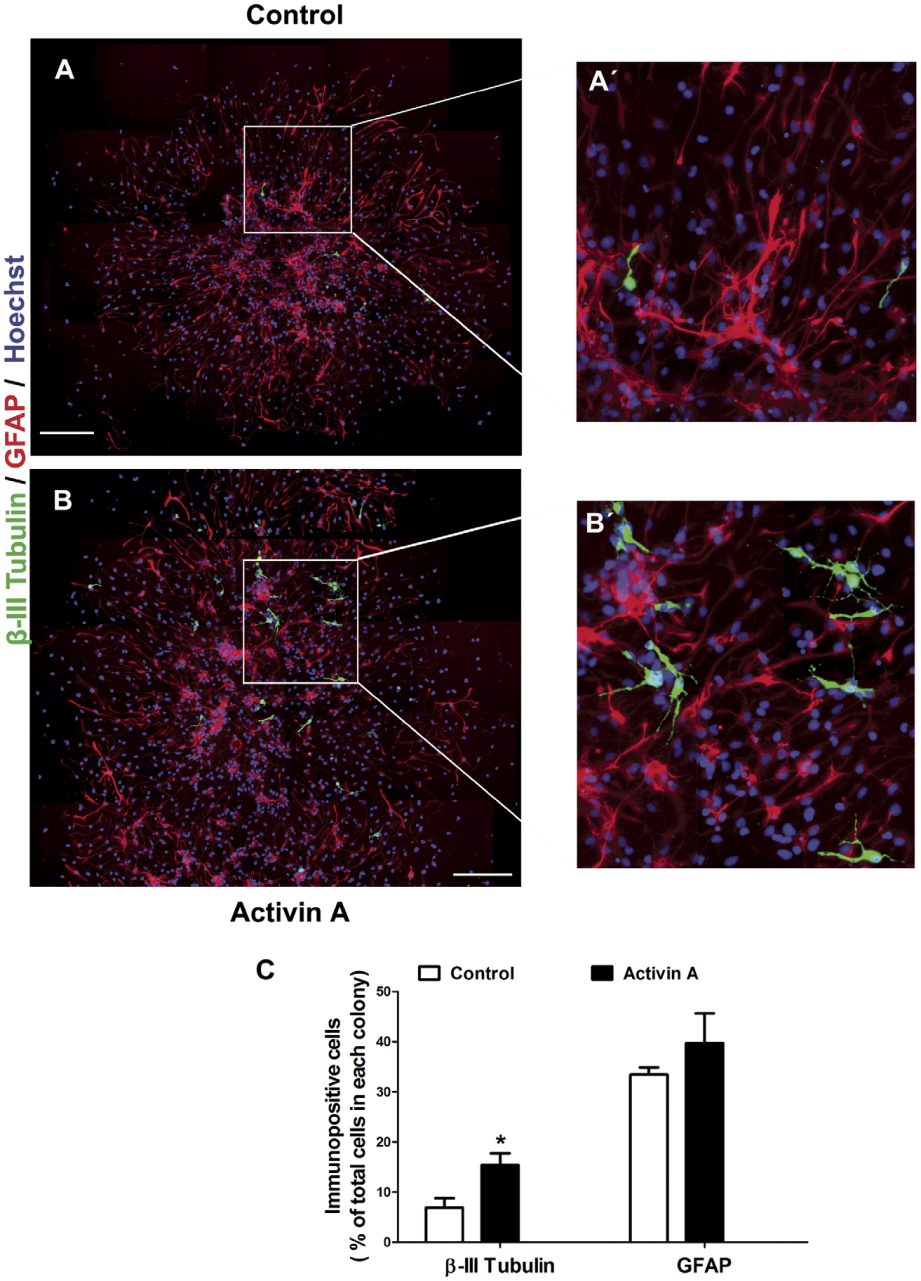
Activin A increases neuronal differentiation in clonal NPC cultures. Cells were grown at low density and the areas with isolated cells were identified with circles with the aid of a marking objective. Cultures were maintained with FGF2 during 6 days and differentiated by 8 additional days. Cells received continuous treatment with Activin A and were immunocytochemically stained for β–III Tubulin and GFAP. **A:** In controls, a few differentiated neurons were found, whereas Activin A (**B**) increased the number of β–III Tubulin^+^ cells. **A’** and **B’** are magnifications of control and Activin-treated cells, respectively. **C:** Quantification of the percentage of neuronal (β-III Tubulin-positive) or astrocytic (GFAP-positive) cells relative to total cell number in each clone. Results are mean ±S.D. *p<0.05 versus control condition. Scale bar = 100 µm.

To test the neurogenic effect of Activin A at a single cell level, we decided to perform experiments at clonal density, in order to determine if this treatment can modify the proportion of differentiated neurons. We plated cells at low density, identified isolated cells with a marking objective and analyzed the differentiated cells that each clone gave rise to after 8 days of differentiation, detecting cells positive for β-III Tubulin or GFAP. In control conditions, neuronal cells were clustered in the center of the colonies, while astroglia was predominantly found in the periphery (Figures 6A and 6A ´). Treatment with Activin A significantly increased the number of neurons present in each clone (Figures 6B and 6C), relative to control conditions, with β-III Tubulin^+^ cells distributed throughout the colonies; GFAP-positive cell numbers were unmodified by Activin A (Figure 6C).

### Antagonism of Activin Type I Receptors Abolishes Neuronal Differentiation in Cerebrocortical Neural Progenitor Cells

**Figure 7:**
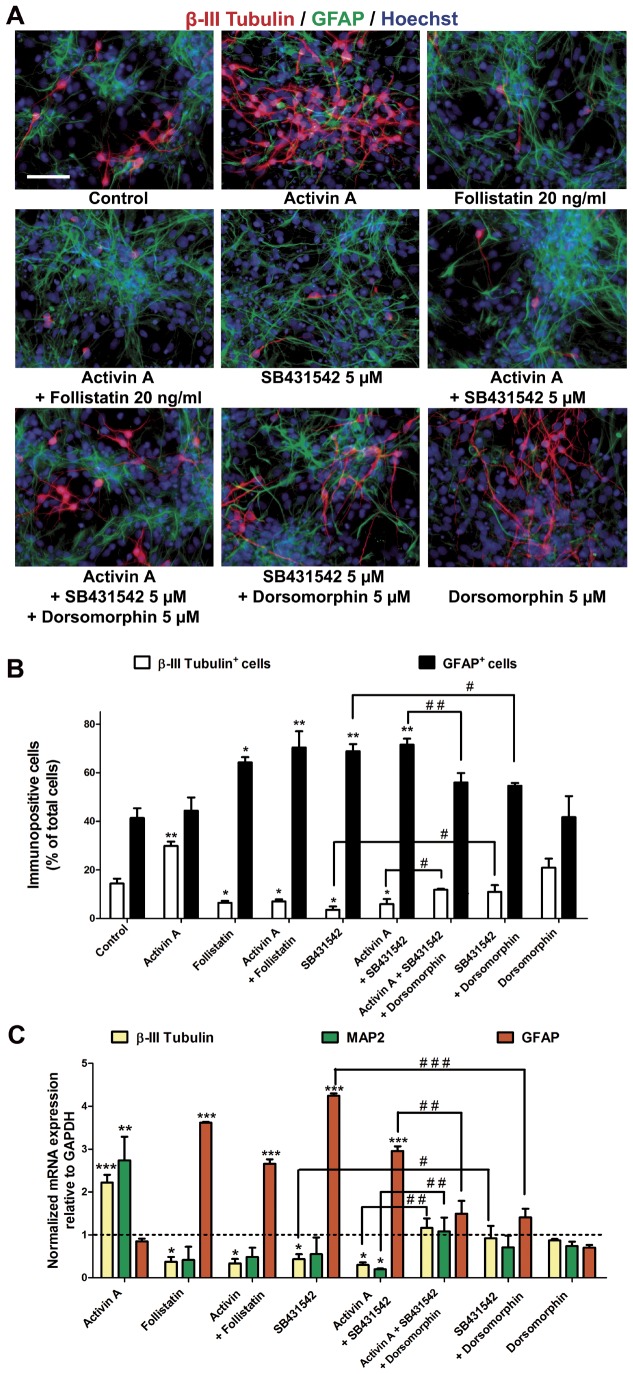
Blockade of Alk receptor activation abolishes neuronal differentiation due to Activin A treatment. Cells were cultured 4 days with FGF2 and 6 days without FGF2. Neuronal and astrocytic differentiation was analyzed by immunocytochemistry and quantitative RT-PCR. Addition of growth factors and inhibitors was made every other day. SB431542 (Alk4, Alk5 and Alk7 inhibitor, 5 µM), Follistatin (Activin A ligand-trap, 20 ng/ml) or Dorsomorphin (BMP antagonists, 5 µM) were added to NPC cultures 1 hour previous to Activin A. **A:** Representative micrographs showing the antagonistic effect of SB431542 or Follistatin on neuronal differentiation caused by Activin A; either of these compounds had the same antagonistic effects in the absence of Activin A, and also increased astrocyte differentiation. Dorsomorphin by itself did not change neuronal/glial proportions relative to controls. Co-treatment with Dorsomorphin and SB431542, however, prevented SB-induced astrocyte differentiation. **B:** Quantification of the total number of β-III Tubulin-positive or GFAP-positive cells in experiments performed by duplicate from 3 independent cultures. In addition to antagonizing the neurogenic effects of Activin A, SB431542 and Follistatin by themselves, decreased the number of neurons and also increased astroglial differentiation. When cells received co-treatment with both inhibitors (Dorsomorphin and SB431542) the proportion of neurons was similar to untreated cells, while the number of astrocytes was not as high as that obtained with Activin A and SB431542. **C:** Real time RT-PCR from cells treated with Activin A plus inhibitors. The represented values were normalized by GAPDH expression level and represented relative to the control condition, set to 1. Co-treatment with Activin A and SB431542 or Activin A plus Follistatin decreased to the half mRNA expression for neuronal markers, while increased GFAP expression. Combined treatment with Dorsomorphin plus SB431542 have neuronal and astrocytic transcript indistinguishable from control values. Results are means ±S.D. The Student-Newman-Keuls was used as a post-hoc test after one-way ANOVA. *p<0.05, **p<0.01 and ***p<0.001 versus control condition. ^#^ *p<0*.05, ^# #^ *p<0*.01, ^###^ *p<0*.001 versus the indicated conditions. Scale bar = 50 µm.

Since type I receptor phosphorylation is necessary for Activin signaling, we wanted to pharmacologically block its signal and assess whether neuronal differentiation is impaired. We used a compound that blocks the activation of TGF-β type I receptor in the presence of its ligand. We also employed a high-affinity antagonist that impedes the binding of Activin A to their receptors, Follistatin. Cells received continuous treatment with the inhibitors 1 hour prior to treatment with 3 ng/ml Activin A. After quantification of immunocytochemistry for β-III Tubulin^+^ and GFAP^+^ cells, we observed a significant two-fold increase in differentiated neurons without changes in astrocytic cells after Activin A addition. This neurogenic effect was sensitive to Follistatin or SB431542, and these inhibitors caused a significant 3-fold decrease in neurons, even when they were added alone (Figure 7A and 7B). Interestingly, astroglial differentiation was increased two-fold after treatment with either Follistatin or SB431542, regardless of Activin A presence (Figure 7A and 7B). The decreased number of neurons suggests an endogenous effect by Activin A, or another member of the family present in the cultures, to drive neuronal differentiation.

Since the results with these inhibitors were highly similar to those obtained after treatment with BMP4, an alternative scenario is that after a blockade in Activin signaling, BMP4 or a similar molecule, promotes glial differentiation. To directly test this idea, we decided to inhibit both BMP and TGF-β pathways by co-treatment with the pharmacological inhibitors Dorsomorphin and SB431542, respectively. Inhibitors were added to cells one hour before treatment with Activin A. Addition of Activin A, Dorsomorphin and SB431542 resulted in differentiation indistinguishable from control conditions, suggesting that Dorsomorphin counteracted SB431542 effect on reducing neurogenesis and augmenting astrogliogenesis (Figure 7A and 7B). Accordingly, GFAP immuno-positive cells were lower than SB431542 with or without Activin A (Figure 7A and 7B). Dorsomorphin alone did not modify neuronal nor astrocytic differentiation.

The results above described were complemented by quantitative RT-PCR analysis. We observed similar induction in β-III Tubulin and MAP2 mRNAs after Activin A treatment, that were significantly reduced by addition of Follistatin or SB431542, in the presence or absence of Activin A, to levels even lower than controls. Follistatin or SB431542 also significantly up-regulated GFAP transcripts. The effects of SB431542 decreasing neuronal and increasing astrocyte transcripts were abolished by Dorsomorphin, regardless of Activin A stimulation (Figure 7C).

## Discussion

This study demonstrates for the first time that Activin A can drive the differentiation of cerebrocortical NPC towards a neuronal phenotype. The increases in neuronal differentiation were still observed when cells were cultured during prolonged periods of time, strengthening the notion that this effect was not due to an early cell cycle exit of neuronal precursors. Activin-induced neuronal differentiation was present in clonal experiments, suggesting an instructive action. We further established that Activin A requires activation of its receptors in order to exert its neurogenic effect, since their pharmacological inhibition abolishes neuronal differentiation. Our results also suggest that, when Activin signaling is precluded, endogenous release of BMP cause decreased neuronal differentiation and enhanced astrogliogenesis.

The observed morphological changes after treatment of cortical NPC with Activin A/TGF-β1 were correlated with subsequent differentiation to neurons. BMP4, on the other hand, caused preferential differentiation to astrocytes, and induced, especially at higher concentrations, the expression of smooth muscle actin. Recently, it was reported that BMP4 caused flattening of cortical NPC, which was associated with dormancy [21]. In agreement with our data, TGF-β1 produced a reduction in cell bodies of hippocampal and cortical mouse NPC without causing loss of Nestin expression [19]. Similar changes (i.e. cell area retraction for neurogenesis and cell surface increase for smooth muscle differentiation), have been reported, although not systematically quantified for neural crest stem cells [25]. Interestingly, for these latter cells, BMPs caused neuronal commitment and the appearance of SMA-positive cells, highlighting the fact that stem cells from different neural origins posses particular responses to these cytokines. The present set of results indicates that although Activin A, TGF-β1 and BMP4 can alter the morphology of proliferating NSC, they do not induce massive premature differentiation, possibly because they cannot overcome the anti-differentiation effects of FGF2.

Activin A drives neurogenesis of NPC both in the presence, but more robustly in the absence of the mitogenic factor FGF2. In proliferating NPC, Activin induced discrete but significant increases in the proportion of neurons. It has been reported that Smad proteins are negatively regulated by MAP kinases (the intracellular effectors of FGF signaling), through phosphorylation of the linker region in R-Smads [31]. In this scenario, FGF2 might attenuate Activin A effects. To observe Activin-induced neurogenesis, this cytokine needs to be present in both proliferation and differentiation phases (Figure S3). Under this continuous treatment with Activin A, we did not find changes in cell proliferation or in apoptotic death, pointing out that, instead of being cell cycle regulator or a survival factor for immature neurons, Activin might have an instructive role on NPC.

TGF-β superfamily is divided into two branches: the BMP/Growth differentiation factors and the TGF-β/Activin. Whereas BMP2/4 inhibit neurogenesis and stimulate the formation of astrocytes [22], [32], [33], [34], [35], [36], [37], TGF-β1 presents neurogenic effects, involving inhibition of proliferation in cultured cortical and hippocampal progenitors [19], [24]. Furthermore, deletion of *Tgf-β2* and *Tgf-β3* in mice results in enhanced proliferation and reduced neurogenesis in telencephalon and mesencephalon [19], [38]. It has also been reported that TGF-β signaling participates in differentiation of the midbrain: TGF-β1-treated neural stem cells from this region showed premature neuronal differentiation through up-regulation of the cell cycle inhibitor protein p27^kip1^ [20]; TGF-β1, acting synergistically with FGF8 and Sonic hedgehog, induced the dopaminergic neuronal markers Nurr1 and Tyrosine Hydroxylase, in ventral midbrain neurospheres [39]. Accordingly, we showed that TGF-β1 had a neurogenic effect in cerebrocortical NPC, while BMP4 had a positive effect in the differentiation into glial cells.

According to an instructive role, in extended differentiation periods, Activin A induced further increases in β–III Tubulin^+^ and MAP2^+^ cells, whereas TGF-β1, a cytokine that causes neuronal differentiation through an early cell cycle exit of neuronal precursors [20], did not elicit further increases in neurons. In agreement, neuronal-specific transcripts were up-regulated by Activin, but not by TGF-β1. Moreover, clonal cultures continuously treated with Activin also showed an increase in the proportion of neurons in clonal-derived colonies, supporting an instructive neurogenic role for Activin in NPC. Interestingly, Activin A did not preclude astroglial differentiation, similar to a previous study reporting a neurogenic role for TGF-β1 in hippocampal and cortical NPC [19], strengthening our hypothesis about the neurogenic action of Activin A.

Although there is evidence that Activin A has neuroprotective effects in neuronal cultures [7], [9], [40], [41], we could not observe any changes in the levels of apoptosis. However, we cannot exclude that Activin may have an early effect on NPC survival, because the TUNEL assay measures cell death at its final stage and we performed this quantification late in the differentiation process. Therefore, if Activin A exerts any neuroprotective effect during proliferation, or at prior stages of differentiation, an earlier marker of apoptosis like activated caspase 3 should be informative.

We show that activation of Activin receptors is important for neuronal differentiation of NPC *in vitro*. Pharmacologically interfering with Activin signaling, abolished the increase in neuronal number and the transcriptional up-regulation of the neuronal markers β-III Tubulin and MAP2. Intriguingly, we found that SB431542 or Follistatin caused a significant decrease in neuronal differentiation, relative to controls, when incubated alone, implying that endogenous Activin signaling plays a role in the differentiation of cultured NPC. Supporting this possibility, transcripts for Activin A, Activin B and TGF-β1-3 have been detected in cortical NPC [19], [21]. Co-incubation of SB431542 with Dorsomorphin, regardless of Activin A addition, restored both neuronal and astroglial differentiation to control levels, suggesting a potential competition between the two TGF-β superfamily branches, which might determine neurogenic or gliogenic differentiation of NPC not only *in vitro*, but also *in vivo,* because these cytokines are also present in brain development.

The results from this study can be correlated with Activin actions *in vivo*. During cerebral cortex formation, Activin A and its receptors are enriched in postmitotic neurons of the deep layers [13], although a neuronal differentiation effect was not reported. In addition, genetic disruption of TGF-β type II receptors in the developing forebrain, showed persistent phosphorylation of Smad 2/3, suggesting Activin as the responsible molecule for these observations [42]. Moreover, mice with postnatal overexpression of Follistatin, driven by the neuronal forebrain αCaMKII promoter, presented increased anxiety-related behavior and decreased neurogenesis in the adult hippocampus [15]. According with an *in vivo* role in adulthood, Activin participates in inducing NSPC proliferation during adult neurogenesis in response to excitotoxic damage, and similar to our results Follistatin infusion after such brain damage produced an inhibitory effect on adult neurogenesis [43].

In summary, our findings reveal that Activin A induces neuronal differentiation of cortical NPC *in vitro*. Blockade of Activin signaling markedly reduced the resulting neuronal population, while promoted astroglial differentiation, suggesting a relevant role of this cytokine for specifying cell fate in NPC. Thus, manipulation of Activin A signaling could be used as a new approach to achieve controlled differentiation of NPC cultures.

## Materials and Methods

### Ethics Statement

Manipulation of the rats used in this study was carried out following the approval of Instituto de Fisiología Celular Animal Care and Use Committee.

### Cell Culture

Isolation of NPC cultures from the forebrains of E14 Wistar rat embryos was performed as previously described [44], [45]. The isolated cells were seeded at 1−1.5×10^6^ in 10-cm diameter culture dishes previously treated with 15 µg/ml poly-L-ornithine (Sigma), and 1 µg/ml human Fibronectin (Invitrogen) in N2 medium (DMEM/F12 1:1, supplemented with 25 µg/l of human insulin, 30 nM sodium selenite, 100 µM putrescine, 20 nM progesterone and 100 mg/l Apotransferrin) with 10 ng/ml of Fibroblast growth factor 2 (FGF2; Peprotech) to promote proliferation, and were incubated at 37°C in a 5% of CO_2_ environment. FGF2 was added daily and N2 medium was changed every other day. The initial plating was considered passage (P)0, and when cells reached 80% confluence they were detached and re-plated to P1 and P2. All reported experiments were performed in P2 cells that were maintained for 4 days in control (N2 medium + 10 ng/ml FGF2) and experimental conditions (N2 medium +10 ng/ml FGF2+ various concentrations of Activin A). For immunocytochemistry, crystal violet, MTT, trypan blue exclusion and TUNEL assays, cells were seeded at 1×10^4^/well onto 12 mm coverslips in 24-well plates (Corning) in control and experimental conditions. Differentiation was promoted by removing FGF2 and keeping the cells for 6, 8 or 10 days in N2 medium plus 200 µM ascorbic acid to promote cell survival after FGF2 withdrawal, in the presence or absence of the tested cytokines. Unless otherwise stated, addition of Activin A (Peprotech) was performed every two days, during proliferation and differentiation phases. In addition, we used as positive controls BMP4 (Peprotech) and TGF-β1 (Peprotech) because their effects on NPC have been previously described [17], [20], [24], [35], [46], [47].

The inhibitor of TGF-β type I receptor kinase, SB431542 (Tocris), was used to establish if the effect of Activin A on neuronal differentiation was mediated by type I receptor activation. SB431542 was used at 5 µM, as inhibitor of kinase activity for Alk4, Alk5 and Alk7; Alk4 is the type I receptor responsible for Activin A signaling, whereas Alk5 is involved in TGF-β1 signaling. In addition, for selectively blocking Activin A effects, we used the ligand trap Follistatin at 200 ng/ml (Peprotech). SB431542 as well as Follistatin were added to cells one hour prior to treatment with 3 ng/ml Activin A during proliferation.

### Immunoprecipitation and Western Blot

Cells at day 4 of proliferation were lysed with TNTE buffer (50 mM Tris–HCl, pH 7.4, 150 mM NaCl, and 5 mM EDTA containing 0.5% Triton X-100) supplemented with proteases (Complete, Roche) and phosphatases (Phospho-stop, Roche) inhibitors. Proteins were obtained by centrifugation at 13 800 g at 4°C for 15 min, and quantified by a modified Bradford assay (Bio-Rad Protein assay, Bio Rad). One mg of total protein was immunoprecipitated (IP) with 1 µg of specific Smad 2/3 antibody (Santa Cruz Biotechnology) overnight, followed by 2 h incubation with protein G sepharose (Fast Flow, Upstate), as previously reported [48], [49]. Immunoprecipitates were separated on 8% sodium dodecyl sulfate–polyacrylamide gel electrophoresis and transferred to nitrocellulose membranes (Amersham Bioscience, USA) which were blocked with 5% non-fat dry milk and incubated overnight with primary antibodies. Pre-stained markers (Invitrogen) were included for size determination. The antibodies used were: Phospho-Smad 2 (Ser465/467) (1∶1000, Cell signaling technologies), Phospho-Smad 3 Ser423/425 (1∶1000, Cell signaling technologies), and Smad 2/3 (1∶100, Santa Cruz Biotechnology). Membranes were washed and incubated with corresponding horseradish Peroxidase-coupled secondary antibodies (1∶10 000, Santa Cruz Biotechnology). Immuno-reactive bands were detected using enhanced chemiluminescence method (ECL kit from Amersham Life Sciences) and developed on photographic film. Smad 2/3 was used as a control for protein loading.

### RNA Extraction, RT-PCR and Real Time RT-PCR

Total RNA was isolated from cells maintained 4 days either in proliferation or differentiation conditions using TRIZOL (Invitrogen). For RNA extraction, cells were seeded at 3×10^5^ in 6-well plates (Corning). Total RNA (500 ng) was treated with DNAse I (invitrogen) and reverse-transcribed to cDNA with oligodT (Invitrogen). The synthesized cDNA was used as substrate for semi-quantitative RT-PCR and for real-time RT-PCR with specific oligonucleotides (Table S2).

For amplification of cDNA encoding for type I and type II receptors of Activin, the cDNA obtained of the RT reaction of proliferating cells was used in PCR containing 2 U Taq DNA polymerase (Invitrogen), 20 pmol of specific primers (Sigma), 500 µM deoxynucleosidetriphosphates and 1.5 mM MgCl2 (for Alk4 and GAPDH) or 2 mM MgCl2 (for ActRII and ActRIIB). The PCR-amplification parameters applied were as follows, for Alk4: denaturalization at 95°C for 15 min, 30 cycles of denaturalization at 95°C for 1 min, annealing at 57°C for 1 min, and elongation at 72°C for 1 min. For ActRII and ActRIIB: denaturalization at 95°C for 15 min, 30 cycles of denaturalization at 95°C for 1 min, annealing at 60°C for 1 min, and elongation at 72°C for 1 min. Final extension at 74°C for 10 min was terminated by rapid cooling at 4°C. PCR products were analyzed in 2% agarose gel electrophoresis and the size of the reaction products was determined by comparison with molecular weight standards after ethidium bromide staining. Reactions with RNA in the absence of retrotranscription were used as negative controls, and no amplifications were found under such conditions (data not shown).

β-III Tubulin, MAP2 and GFAP. PCR reactions were performed using 2.5 U of recombinant Taq Polymerase (Invitrogen), 0.4 mM of each oligonucleotide and dNTPs, and 1.5 mM MgCl2 diluted in the buffer recommended by the manufacturer (50 mM KCl, 20 mM Tris–HCl, and pH 8.4) and 5 µM Syto 9 (Invitrogen). Quantitative analysis of cDNA amplification was performed using the rotor gene 6000 system (Corbett Life Science, San Francisco, CA). Melting curves were performed for each reaction to ensure that only one product was present. Glyceraldehyde 3-phosphate Dehydrogenase (GAPDH) was used as a normalization control for all reactions. The reported value was further normalized to the control condition [50].

### Immunocytochemistry

Cortical cells were fixed at day 4 of proliferation or at day 6,8 or 10 of differentiation with 4% paraformaldehyde in PBS, pH = 7.4 for 20 min at 4°C, permeabilized and blocked as it was described above. Cells were incubated overnight at 4°C with the following primary antibodies, diluted in PBS containing 10% NGS: rabbit polyclonal anti-β-III Tubulin (1∶2000, Babco Covance); rabbit polyclonal anti-Glial Fibrillary Acidic Protein (GFAP; 1∶2000, DAKO); mouse monoclonal antibody anti-O4 (1∶400, Chemicon); mouse monoclonal antibody anti-Microtubule associated protein 2 (MAP2; 1∶500, Chemicon); mouse monoclonal anti-Nestin (1∶100; Developmental Studies Hybridoma Bank; 1∶1000, Covance); Smad 2/3 (1∶100, Santa Cruz Biotechnology). Then, cells were incubated for 1–2 hours with the secondary antibodies Alexa-Fluor 488 anti-rabbit IgG, Alexa 568 anti-mouse IgG (1∶1000; Molecular Probes), Alexa 568 anti-mouse IgM (1∶1000, Invitrogen), Alexa 488 anti-mouse IgG (1∶1000, Molecular Probes), Alexa 568 anti-rabbit IgG (1∶1000, Molecular Probes) diluted in PBS/10% NGS. Nuclei were identified by staining with Hoechst 33258 (1 ng/ml; Sigma). Immunostainings were analyzed with an epifluorescence microscope (Nikon, Eclipse TE2000-U) and photographed with a Nikon digital camera (DMX1200 F). Cells without being treated with primary antibodies were used as negative control, where no unspecific staining was observed.

### Phalloidin Staining and Cell Area Determination

Actin cytoskeleton of proliferating cortical NPC was studied through phalloidin staining. Cells were fixed at day 4 of proliferation with 10% formol in PBS, pH = 7.4 for 10 min at room temperature, permeabilized and blocked for 30 minutes with 0.1% Triton X-100 and 10% NGS in PBS. Fixed cells were incubated 30 minutes with Phalloidin coupled to Alexa Fluor 488 (Molecular Probes) diluted in PBS according to manufacturer instructions. Nuclei were counterstained with Hoechst 33258 (1 ng/ml). Epifluorescence pictures were also obtained as above described. Cell area was determined from pictures taken with a Nikon digital camera using the free Image Tool 3.00 UTHSCSA software. Cell area measurements were performed by determining the cell area in the cells stained by Phalloidin in at least 10 random fields in duplicate, from 3 independent experiments.

### Crystal Violet Assay

The total number of viable cells was determined using the crystal violet assay, in which optic density is correlated with the amount of viable cells. Cells were fixed in 10% formol-PBS, pH 7.4, washed and incubated with a 0.5% solution of crystal violet for 10 min at 21°C. After thorough washing with bi-distillated water, acetic acid (33% vol:vol in H_2_0) was added to elute the dye. The optical density was measured at 595 nm using a spectrophotometer (Beckman DU650), and the results are expressed as percent with respect to control conditions [45].

### MTT Cell Viability Assay

The MTT assay was used for determining cell viability in proliferating and differentiating cells according to the protocol described by Mosmann [51]. Briefly, at day 4 of proliferation or day 6 of differentiation, cells were incubated for 3 hours with 500 ng/ml Thiazol blue Tetrazolium bromide (MTT; Sigma). The insoluble product of MTT reduction was dissolved with acidified isopropanol and the absorbance was measured at 570 nm using a spectrophotometer (Beckman DU650). Measurements are expressed as percent in relation to control conditions.

### Trypan Blue Exclusion Assay

Trypan blue is a polar dye that is excluded from viable cells and penetrate the damaged membranes, marking dead cells. In order to determine the number of viable cells, trypan blue exclusion assays were performed at day 4 of proliferation or day 6 of differentiation. Cells were detached of the plates by trypsinization (0.05% trypsin-EDTA, Gibco), trypsin action was neutralized by incubation with DMEM with 10% fetal bovine serum, cells were centrifuged and re-suspended in N2 medium. The resulting cell suspensions were mixed with 0.4% trypan blue solution (Sigma) and counted in a hemacytometer. Cells excluding the dye were considered viable, whereas blue cells were classified as dead cells. Measurements are expressed as percent of viable cells in relation to total cells (viable + dead).

### Bromodeoxyuridine (BrdU) Incorporation Assay

For cell proliferation analysis, cells treated with FGF2 were incubated for 3 h with 10 µM 5-bromo-2′-deoxyuridine (BrdU, Roche), washed with fresh medium supplemented with FGF2 and fixed 21 h later. After fixation with 4% paraformaldehyde in PBS, pH = 7.4, for 20 min at 4°C, cells were incubated with 1 N HCl for 30 min at 25°C, and neutralized by washing with 0.1 M borate buffer, pH = 8.5. Preparations were blocked for 1 h with 0.3% Triton X-100 (Sigma) and 10% normal goat serum (Microlab, Mexico) in PBS. Cells were incubated overnight at 4°C with a monoclonal rat anti-BrdU antibody (1∶100, Accurate) followed by three washes with 1% BSA/PBS. Secondary antibodies were added (Alexa Fluor 488 goat anti-rat IgG; Molecular Probes) at 1∶1000 dilution in blocking solution without Triton for 1 h at 25°C in the dark, and washed three times with PBS. Nuclei were stained with Hoechst 33258. Immunostainings were visualized and photographed as described before. Negative controls did not show unspecific staining.

### TUNEL Assay

NPC in P2 were plated onto 12 mm coverslips, and treated every other day with Activin A, TGF-β1 and BMP4, or maintained in control conditions. Cells were fixed with 4% paraformaldehyde after 6 days of differentiation, and then washed 3 times with PBS pH = 7.4. For detection of apoptotic cells, the Terminal deoxy-transferase-mediated dUTP nick-end labeling (TUNEL, In Situ Cell Death Detection Kit, Roche Diagnostics) was used to evaluate the effect of cytokines on cell death according to manufactureŕs instructions. Apoptotic cells were analyzed using fluorescence microscopy.

### Clonal Analysis

NPC at P2 were seeded at low density (1 000 cells in 6-cm plates) and after 12 h of plating when most cells are attached into the plates, well isolated single cells were marked with a 3-mm circle (Nikon) on the bottom of the plate. Cells were maintained during 12 days (6 days in proliferative conditions and 6 days in differentiation conditions) and receiving treatment with Activin A every two days. After differentiation, cell types in clones were analyzed by double-staining with combinations of antibodies for neurons and glia (β-III Tubulin and GFAP, respectively).

### Cell Counting

Cell counts from BrdU, immunocytochemistry and TUNEL experiments were performed from pictures taken with a Nikon digital camera, analyzed with the Nikon ACT-1 imaging software. Quantification of cells was performed by using the ImageJ 1.34s program to count the number of Hoechst-stained nuclei (total cells) and the specified markers in at least 10 random fields in duplicate, from at least 4 independent experiments.

### Statistics

All data are presented as mean ± standard deviation (S.D.). One-way ANOVA was performed for statistical analysis. The post-hoc Student-Newman-Keuls test was used for multiple comparisons between treated and control groups and values of P *<*0.05 were considered as statistically significant. Graphs were generated using GraphPad Instat software.

## Supporting Information

Figure S1 **Cultured neural progenitor cells do not express differentiation markers in the presence of FGF2 (proliferation) and are multipotent, generating neurons, oligodendrocytes and astrocytes after differentiation.** **A**) Cells were fixed and immuno-stained for Nestin/Glial Fibrillary Acidic Protein (GFAP) or for Nestin/Smooth Muscle Actin (SMA). No signal was detected for GFAP and SMA, ruling out contamination with differentiated cells. **B**) After FGF2 withdrawal, differentiated cells express markers for neurons (β-III Tubulin), oligodendrocytes (O4) and astrocytes (GFAP).
(TIF)

Figure S2 **Activin A and TGF-β1 induce a small but significant increase in the proportion of differentiated neurons in the presence of FGF2, relative to control conditions.** The quantification is presented in Figure 2E.
(TIF)

Figure S3 **Treatments with Activin A or TGF-β1 only during proliferation with FGF2, or only in differentiation phase, do not increase the number of neurons.** Cultures were analyzed 6 days after FGF2 removal. Quantification of the percentage of neuronal (β-III Tubulin or MAP2-positive) or astrocytic (GFAP-positive) cells relative to total cell number after treatment with 3 ng/ml Activin A, 0.5 ng/ml TGF-β1 or 5 ng/ml BMP4. Experiments were performed in duplicate, and pictures taken from ten fields from three independent experiments were considered. Neither Activin A nor TGF-β1 increased neuronal differentiation when they were added only during proliferation (**A**) or differentiation (**B**) phases, whereas BMP4 significantly decreased the number of neurons and increased astrocytogenesis. Results are mean ±S.D. *P<0.05, **P<0.01 and ***P<0.001 versus control condition.
(TIF)

Figure S4 **Continuous treatment with BMP4 has dose-dependent effects in the differentiation of NSPC. Cells were maintained 10 days in culture (4 days in proliferative and 6 days in differentiation conditions) and received continuous treatment with BMP4 ascending concentrations.** **A**) Representative micrographs of the labeling for GFAP (green) and Smooth Muscle Actin (SMA, red) and nuclear detection by Hoechst (blue), showing the effect of 20 ng/ml BMP4 treatment on the percentage of GFAP- and SMA-positive cells. At this concentration, BMP4 induced a high proportion of SMA+ cells. **B**) Quantification of the percentage of astrocytic (GFAP-positive) or smooth muscle (SMA-positive) cells relative to total cell number. Cell counts were performed in ten pictures taken from three independent experiments made in duplicate. Results are means ±S.D. *P<0.05 and ***P<0.001 versus control. Scale bar = 50 µm.
(TIF)

Figure S5 **Treatment with Activin A, TGF-β1 or BMP4 does not modify the number of differentiated cells after extended incubation periods.** Cells were continuously treated with Activin A, TGF-β1 or BMP4 for 4 days in the presence of FGF2 and let to differentiate for 6, 8 or 10 days (10, 12 and 14 days in culture, respectively) to quantify the number of nuclei stained with Hoechst at the indicated days from three independent experiment performed by duplicate. No significant differences relative to controls (dotted line) were found in the number of nuclei present in the cultures using ANOVA followed by Student-Newman-Keuls test. Results are shown as mean ±S.D.
(TIF)

Table S1 **Treatment with cytokines in proliferation-only, differentiation-only or proliferation and differentiation does not affect total cell number quantified 6 days after FGF2 removal.** NPC were grown in N2 medium with (proliferation) or without (differentiation) FGF2 with Activin A, TGF-β1 or BMP4 during 4 and 6 days, respectively. Quantification of total cell number by nuclei counting was performed at day 10 of culture. Cells were treated with cytokines during proliferation only, differentiation only, or during both stages (10 days). No significant changes in cell number were found relative to control conditions using ANOVA followed by Student-Newman-Keuls test. Micrographs were taken from ten fields in three independent experiments and results are expressed as mean ±S.D.
(DOC)

Table S2 **Sequence of primers used in RT-PCR and qRT-PCR.**
(DOC)
