# Supplementary figures and images for: Activin A Promotes Neuronal Differentiation of Cerebrocortical Neural Progenitor Cells
Source: PLoS One. 2012 Aug 22;7(8):e43797. doi: 10.1371/journal.pone.0043797 (PMC3425505; doi:10.1371/journal.pone.0043797)

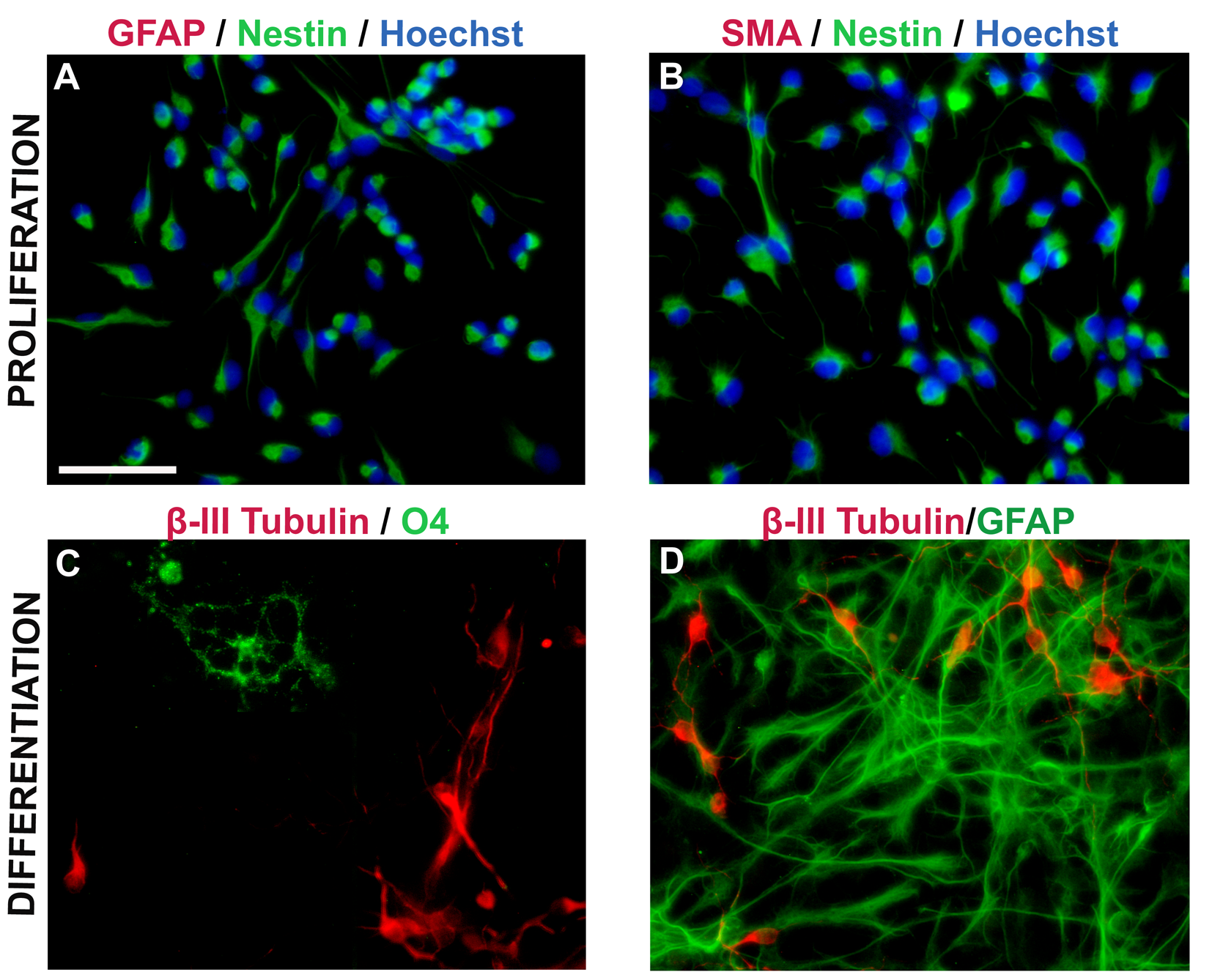

Supplement: Figure S1 — Cultured neural progenitor cells do not express differentiation markers in the presence of FGF2 (proliferation) and are multipotent, generating neurons, oligodendrocytes and astrocytes after differentiation. A) Cells were fixed and immuno-stained for Nestin/Glial Fibrillary Acidic Protein (GFAP) or for Nestin/Smooth Muscle Actin (SMA). No signal was detected for GFAP and SMA, ruling out contamination with differentiated cells. B) After FGF2 withdrawal, differentiated cells express markers for neurons (β-III Tubulin), oligodendrocytes (O4) and astrocytes (GFAP). (TIF) [file pone.0043797.s001.tif]

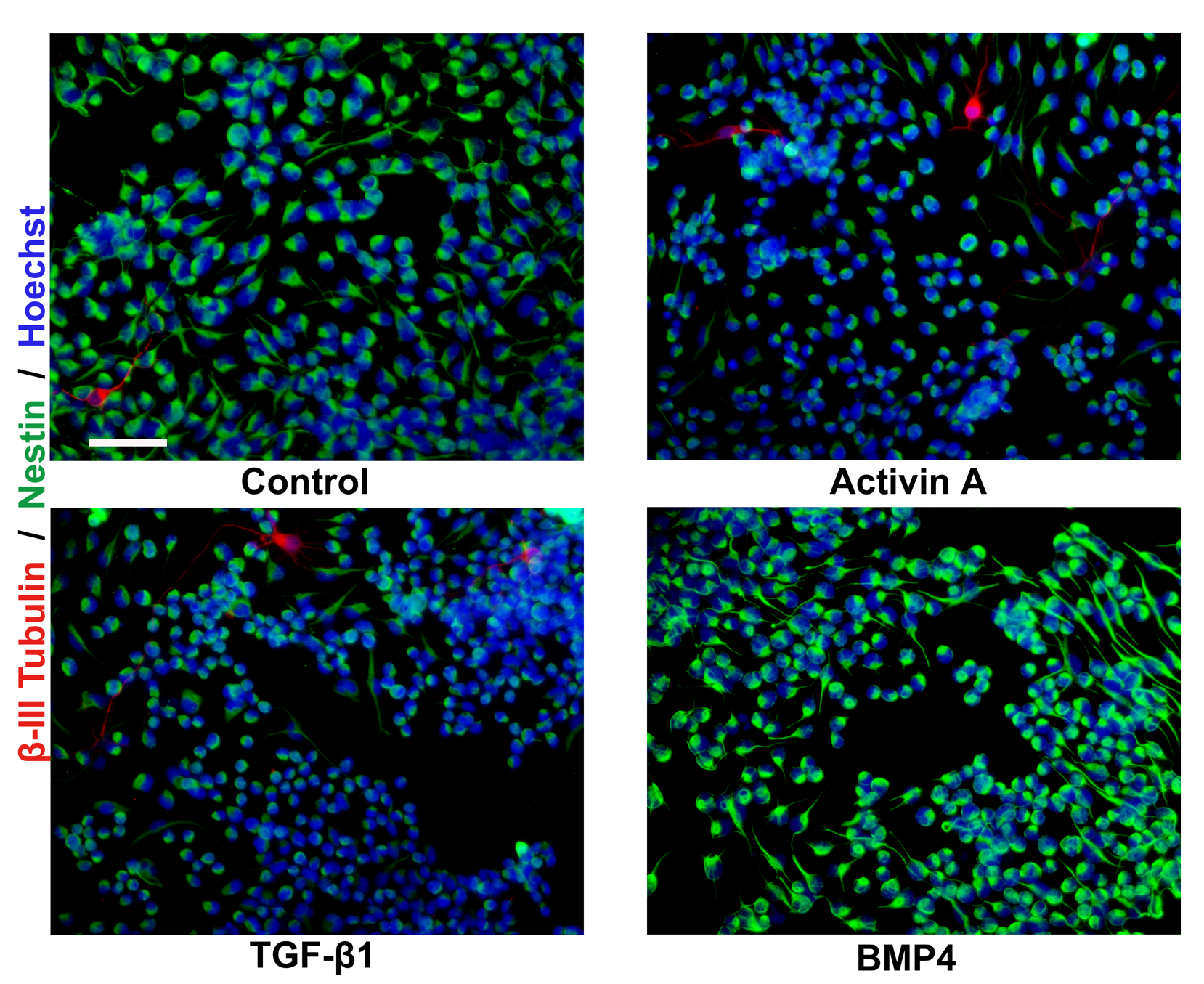

Supplement: Figure S2 — Activin A and TGF-β1 induce a small but significant increase in the proportion of differentiated neurons in the presence of FGF2, relative to control conditions. The quantification is presented in Figure 2E. (TIF) [file pone.0043797.s002.tif]

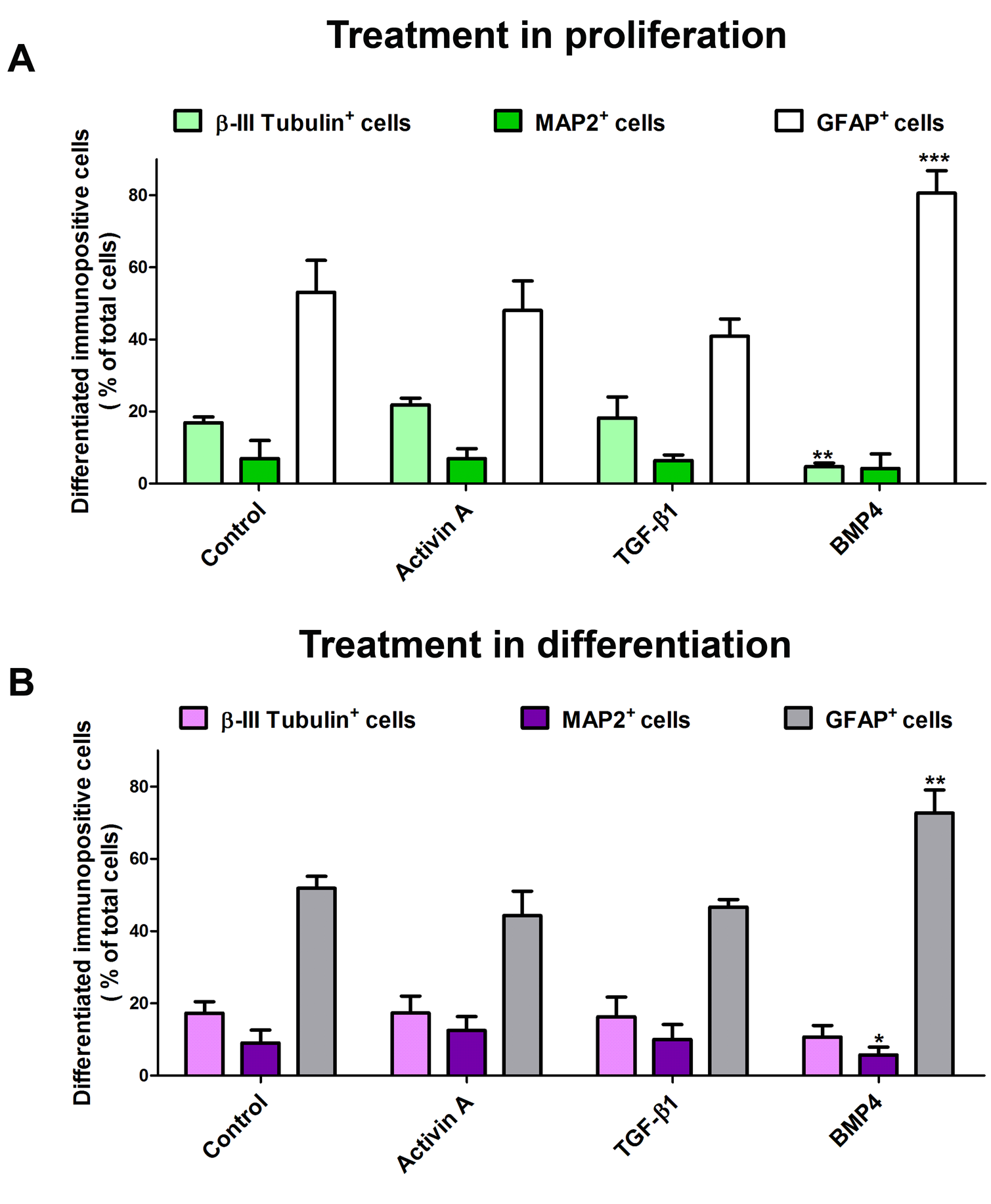

Supplement: Figure S3 — Treatments with Activin A or TGF-β1 only during proliferation with FGF2, or only in differentiation phase, do not increase the number of neurons. Cultures were analyzed 6 days after FGF2 removal. Quantification of the percentage of neuronal (β-III Tubulin or MAP2-positive) or astrocytic (GFAP-positive) cells relative to total cell number after treatment with 3 ng/ml Activin A, 0.5 ng/ml TGF-β1 or 5 ng/ml BMP4. Experiments were performed in duplicate, and pictures taken from ten fields from three independent experiments were considered. Neither Activin A nor TGF-β1 increased neuronal differentiation when they were added only during proliferation (A) or differentiation (B) phases, whereas BMP4 significantly decreased the number of neurons and increased astrocytogenesis. Results are mean ±S.D. *P<0.05, **P<0.01 and ***P<0.001 versus control condition. (TIF) [file pone.0043797.s003.tif]

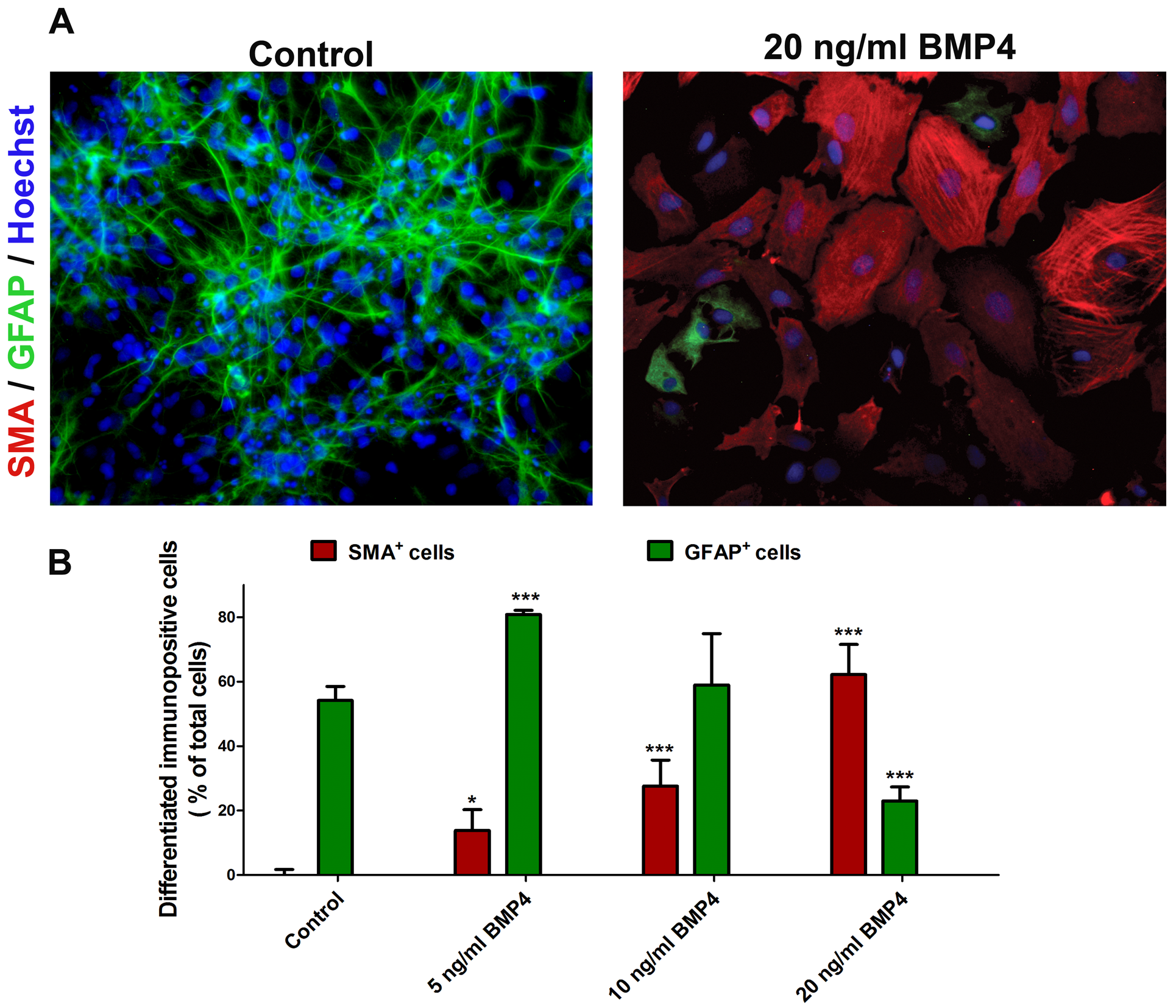

Supplement: Figure S4 — Continuous treatment with BMP4 has dose-dependent effects in the differentiation of NSPC. Cells were maintained 10 days in culture (4 days in proliferative and 6 days in differentiation conditions) and received continuous treatment with BMP4 ascending concentrations. A) Representative micrographs of the labeling for GFAP (green) and Smooth Muscle Actin (SMA, red) and nuclear detection by Hoechst (blue), showing the effect of 20 ng/ml BMP4 treatment on the percentage of GFAP- and SMA-positive cells. At this concentration, BMP4 induced a high proportion of SMA+ cells. B) Quantification of the percentage of astrocytic (GFAP-positive) or smooth muscle (SMA-positive) cells relative to total cell number. Cell counts were performed in ten pictures taken from three independent experiments made in duplicate. Results are means ±S.D. *P<0.05 and ***P<0.001 versus control. Scale bar = 50 µm. (TIF) [file pone.0043797.s004.tif]

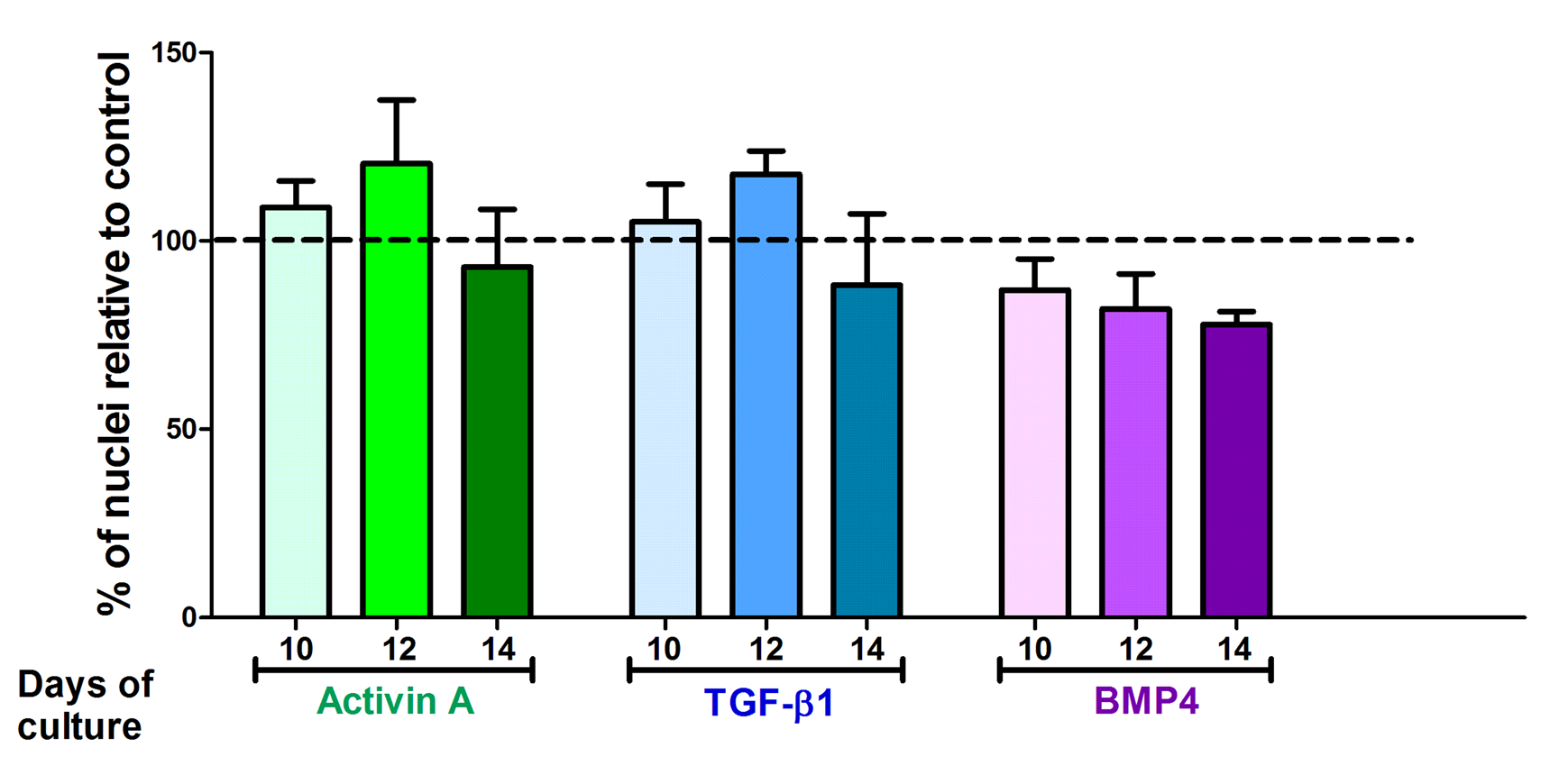

Supplement: Figure S5 — Treatment with Activin A, TGF-β1 or BMP4 does not modify the number of differentiated cells after extended incubation periods. Cells were continuously treated with Activin A, TGF-β1 or BMP4 for 4 days in the presence of FGF2 and let to differentiate for 6, 8 or 10 days (10, 12 and 14 days in culture, respectively) to quantify the number of nuclei stained with Hoechst at the indicated days from three independent experiment performed by duplicate. No significant differences relative to controls (dotted line) were found in the number of nuclei present in the cultures using ANOVA followed by Student-Newman-Keuls test. Results are shown as mean ±S.D. (TIF) [file pone.0043797.s005.tif]
