# Supplementary material for: Activin A Promotes Neuronal Differentiation of Cerebrocortical Neural Progenitor Cells
Source: PLoS One. 2012 Aug 22;7(8):e43797. doi: 10.1371/journal.pone.0043797 (PMC3425505; doi:10.1371/journal.pone.0043797)
Supplement: Table S1 — Treatment with cytokines in proliferation-only, differentiation-only or proliferation and differentiation does not affect total cell number quantified 6 days after FGF2 removal. NPC were grown in N2 medium with (proliferation) or without (differentiation) FGF2 with Activin A, TGF-β1 or BMP4 during 4 and 6 days, respectively. Quantification of total cell number by nuclei counting was performed at day 10 of culture. Cells were treated with cytokines during proliferation only, differentiation only, or during both stages (10 days). No significant changes in cell number were found relative to control conditions using ANOVA followed by Student-Newman-Keuls test. Micrographs were taken from ten fields in three independent experiments and results are expressed as mean ±S.D. (DOC) [file pone.0043797.s006.doc]

**Table S1.** Treatment with cytokines in proliferation-only, differentiation-only or proliferation and differentiation does not affect total cell number quantified 6 days after FGF2 removal.

|  | Treatment given in proliferative conditions | Treatment given in differentiative conditions | Treatment continuously given |
| --- | --- | --- | --- |
| Control | 2931.2 ± 497.8 | 2797.0 ± 322.2 | 2830.3 ± 584.4 |
| 3 ng/ml Activin A | 2972.7 ± 564.5 | 2724.3 ± 495.2 | 2862.0 ± 462.8 |
| 0.5 ng/ml TGF-β1 | 2957.2 ± 411.4 | 2726.7 ± 243.7 | 2707.8 ± 485.0 |
| 5 ng/ml BMP4 | 2939.0 ± 504.1 | 2859.1 ± 610.1 | 2598.8 ± 376.2 |

NPC were grown in N2 medium with (proliferation) or without (differentiation) FGF2 with Activin A, TGF-β1 or BMP4 during 4 and 6 days, respectively. Quantification of total cell number by nuclei counting was performed at day 10 of culture. Cells were treated with cytokines during proliferation only, differentiation only, or during both stages (10 days). No significant changes in cell number were found relative to control conditions using ANOVA followed by Student-Newman-Keuls test. Micrographs were taken from ten fields in three independent experiments and results are expressed as mean ± S.D.
