# Supplementary material for: Activin A Promotes Neuronal Differentiation of Cerebrocortical Neural Progenitor Cells
Source: PLoS One. 2012 Aug 22;7(8):e43797. doi: 10.1371/journal.pone.0043797 (PMC3425505; doi:10.1371/journal.pone.0043797)
Supplement: Table S2 — Sequence of primers used in RT-PCR and qRT-PCR. (DOC) [file pone.0043797.s007.doc]

**Table S2.** Sequence of primers used in RT-PCR and qRT-PCR.

| Name | Right primer  5´ 3´ | Left primer  5´ 3´ | Product size (bp) |
| --- | --- | --- | --- |
| ActRII | AAGTTGGCGTTCGCCGTCTTT | CACAGGTCCACATCAACACTG | 813 |
| ActRIIB | ACTCGGGAGTGCATCTACTAC | CAGCTCGTTCCACGTGATGA | 889 |
| Alk1 | GGGCAAGAGACAAGATCAGC | GCGTTAGGAAGCATTTGGAG | 492 |
| Alk2 | GTTGGCCTTATCATCCTCCTC | ATTCGTGCTCTGGGAATGCAT | 729 |
| Alk3 | GATCAGGGAGAAACCACGTTA | AGCTAACTTGAGTAGGGCTCT | 730 |
| Alk4 | CACGCACTGCTGCTATATTGA | CGACCTTTCGATTTCCTCAA | 1 062 |
| Alk5 | AAGAACAATTGCAAGGACCATT | CTATGGGCAATAGCTGGTTTTC | 395 |
| Alk6 | ATGCTCTTACGACGCTCTGGA | AGGTCGGGCTTCTTTGTCTTTT | 473 |
| BMPRII | AGAGGATGGCTGAACTCATGA | CAAATTTTAGCCGGGGTTCTT | 825 |
| GAPDH | ATCACCATCTTCCAGGAGCG | CCTGCTTCACCACCTTCTTG | 550 |
| TbRII | ACGGCGTTTCATGCTAAGGGCAACC | CCCTCGGGGTCGTGGTCCCAGCACT | 660 |
| qRT-PCR |  | | |
| β-III Tubulin | TGCAGGCAGTCACAATTCTC | GGCCTTTGGACACCTATTCA | 199 |
| GAPDH | GGATGGAATTGTGAGGGAGA | GTGGACCTCATGGCCTACAT | 157 |
| GFAP | GCACACCTCACATCACATCC | GAAGAAAACCGCATCACCAT | 190 |
| MAP2 | TGGGCAAGGGATTTCTACAG | CAAAGAGAAGGTGGCAAAGC | 156 |
